# Detection, Genophenotypic Characterization, and Antimicrobial Resistance of Microbial Contaminants

**DOI:** 10.3390/microorganisms11051350

**Published:** 2023-05-22

**Authors:** Sunghyun Yoon, Sandeep Kondakala, Minjae Kim, Steven L. Foley, Ohgew Kweon, Seongjae Kim

**Affiliations:** 1 Division of Microbiology, National Center for Toxicological Research, U.S. Food and Drug Administration, 3900 NCTR Road, Jefferson, AR 72079, USA; sunghyun.yoon@fda.hhs.gov (S.Y.); sandeep.kondakala@fda.hhs.gov (S.K.); steven.foley@fda.hhs.gov (S.L.F.); 2 Natural Resource Ecology Laboratory, Colorado State University, 711 Oval Drive, Fort Collins, CO 80523, USA; minjaekim45@gmail.com

## 1. Detection and Identification of Microbial Contaminants

Microbial contamination is the inadvertent presence of microbes or their byproducts in materials or environments. The contamination of consumables, such as food, cosmetics, and pharmaceutical products, by pathogenic microbes is of increasing public health concern as the global demand for these products increases. Understanding the risk factors associated with different microorganisms has led to improvements in controlling microbial contamination [1,2]. This Special Issue was established to gather and share high-quality scientific articles on microbial contamination and contains six original research articles covering a range of diverse topics related to microbial contamination [3,4,5,6,7,8]. As revealed from the retrospective analysis of the articles, microbial contamination challenges are often focused around three key issues—the detection, genophenotypic characterization, and antimicrobial resistance (AMR) of microbial contaminants, whereby all of which are interconnected in a pleiotropic and epistatic manner. These three issues are the main scope of this Special Issue, and this editorial aims to promote communication and collaboration among professionals to effectively control microbial contamination.

The detection and identification of microbial entities are crucial in the risk assessment and management of microbial contamination [9]. Different methods and algorithms are used for detecting microbial contaminants in various sample conditions. Conventional culture-based methods have been proven to be reliable, reproducible, and resourceful tools for over a century in determining microbial contamination. The current state-of-the-art detection techniques are faster and exhibit reproducible sensitivity, but no single approach meets all of the emerging criteria for effective and quick results. Therefore, it is essential to select the appropriate strategy for successful safety interventions to limit microbial contamination in different settings. There has been a recent review article that offers a comprehensive overview of microbial detection, covering recent advancements, ongoing challenges, and future directions [9].

Detecting microbial contaminants in samples with low biomass and complex matrices is a challenging task that requires rapid, sensitive, and accurate detection methods for effective safety interventions. In this Special Issue, several articles explore ways to overcome these challenges and improve microbial detection outcomes [3,4,5,6,8].

### 1.1. Microbial Survey of Tattoo Inks with Low Biomass and Complex Nature

The compositions of tattoo inks are complex and varied, containing a range of chemicals, dyes, and preservatives. This complexity can complicate the detection of microbial contaminants. The article by Yoon et al. presents the findings of a microbial survey of 47 sealed and unopened tattoo inks, using the FDA’s Bacteriological Analytical Manual (BAM) Chapter 23 [4]. The study identified potentially pathogenic bacteria present in the tattoo inks, indicating that even when hygienic procedures are followed, contaminated tattoo inks can lead to microbial infections. The BAM Chapter 23 provides guidelines for the microbiological analysis of cosmetic products, including tattoo inks (https://www.fda.gov/food/laboratory-methods-food/bam-methods-cosmetics, accessed on 10 April 2023). The recommended laboratory procedures include the use of direct colony counts and enrichment culturing methods to isolate microbial contaminants from cosmetic products. Dilution and plating media that partially inactivate preservative systems commonly found in tattoo inks are also used in the process to minimize the inhibition of microbial contaminants.

### 1.2. Tracking Pathogens from Pig Production to Pork Meat Distribution Phases

According to the World Health Organization (WHO), microbial contamination in the food industry causes more than 200 different diseases that result in 420,000 annual deaths, as well as significant economic losses (https://www.who.int/news-room/fact-sheets/detail/food-safety, accessed on 10 April 2023). Therefore, ensuring food safety is critical and requires effective microbiological analysis throughout the entire food chain, including production, processing, distribution, and consumer handling.

The article by Bae et al. in this Special Issue provides an analysis of how pathogens are transmitted from pigs and the producing environment to pork meat products throughout meat processing plants [8]. The authors identified a total of 283 presumptive pathogenic bacteria from 126 samples, including Shiga-toxin-producing *E*. *coli* (STEC), *Listeria monocytogenes*, and *Staphylococcus aureus*, and analyzed the isolated bacteria for their antimicrobial susceptibility. Additionally, the authors analyzed the pulsed-field gel electrophoresis (PFGE) patterns of 12 STEC isolates, which suggested that STEC strains found in the pork meat were likely contaminated by workers or the environment in retail stores. These findings are essential for developing practical food safety quality control and monitoring systems. In another study, Bae et al. developed a novel spraying system to address the limitations of conventional poultry farm disinfection methods during production cycles [7]. Conventional methods have high toxicity levels and limited equipment capabilities. The novel system consists of a high-pressure sprayer and pH-neutral electrolyzed water (NEW) disinfectant, producing an optimal size of disinfectant particles. These particles effectively reduced airborne microbes and prevented the transmission of harmful bacteria between experimental chicks in indoor spaces.

### 1.3. Transitioning from Traditional Culture-Based Methods to Molecular-Based Methods in Pharmaceutical Sectors

Medical and industrial sectors face the challenge of detecting microbes quickly and accurately, often relying on traditional culture-based methods that are bulky, expensive, and time-consuming. However, recent research has revealed that some microbes can enter a state of being “viable but not culturable (VBNC)”, making them difficult to detect using traditional culture-based methods [10,11]. Thus, there is a growing need for new, rapid, and efficient molecular-based tools for microbial detection that are suitable for use in pharmaceutical industries and regulatory agencies.

The *Burkholderia cepacia* complex (BCC) is a group of closely related pathogenic *Burkholderia* species that can contaminate non-sterilized pharmaceutical materials and long-term water-based products, posing a potential risk to public health [5,6]. In their articles, Daddy Gaoh et al. have presented two new molecular-based methods for the selective detection of live BCC in different materials: a flow-cytometry-based detection method using a fluorescently labeled oligonucleotide *Kef* probe and a droplet digital PCR (ddPCR) with a variant of propidium monoazide (PMAxx) [5,6]. The study noted that designing BCC-specific primers or probes is challenging due to the close genomic and phenomic relatedness of BCC with other non-BCC strains. The challenge of designing BCC-specific primers or probes was overcome by adopting a pan-genome-based bioinformatics pipeline to analyze complete *Burkholderia* genomes and identify BCC-specific gene clusters that are only present in BCC genomes [5,6]. Additionally, the research group combined PMAxx with ddPCR to specifically detect viable BCC cells, addressing a main disadvantage of molecular-based methods that can lead to false positive results [6]. 

## 2. Genophenotypic Characterization

Genophenotypic characterization is a crucial process in understanding and controlling the spread of microbial contaminants. There are various genotypic characterization techniques such as serotyping, multilocus sequence typing (MLST), PFGE, random amplified polymorphism DNA (RAPD), matrix-assisted laser desorption/ionization time-of-flight mass spectrometry (MALDI-TOF MS), AMR testing, and whole genome sequencing [3,8]. These methods provide important information on the genetic differences that can impact phenotypic features of microbial contaminants, which can help to identify their sources, modes of transmission, resistance mechanisms, and potential health impacts. 

### 2.1. PFGE-Based Tracking of Pathogens from Farm to Distribution

In the study by Bae et al., PFGE pattern analysis was used to track the transmission of STEC from the farm to throughout food processing and distribution [8]. The authors were able to group the isolates based on their sample source and identified four pulsotypes with high similarity in their PFGE banding patterns (≥99% similarity). The study suggested a possible contamination source from either workers or the environment in the retail stores, based on the shared genomic fingerprint pattern that was found in all of the STEC strains in the retail store and contaminated meats. 

### 2.2. Molecular Typing Using MALDI-TOF MS and RAPD Assay of S. aureus Strains 

Similarly, Yoon et al. used multiple genophenotypic characterization methods to analyze *S*. *aureus* strains isolated from stool samples of diarrheal patients [3]. The strains were characterized for their antimicrobial susceptibility, enterotoxin genes, and molecular typing using MALDI-TOF MS and RAPD assays. Molecular typing using both methods indicated that *S. aureus* exhibited diverse clonal lineages, and there were no correlations observed between the profiling of enterotoxin, MALDI-TOF MS, and RAPD. 

## 3. Antimicrobial Resistance 

AMR is a concerning public health issue, as the emergence of resistant bacteria makes infections more difficult to treat and increases the risk of mortality. The overuse and misuse of antibiotics have contributed to the spread of antibiotic-resistant bacteria, which have developed resistance mechanisms for every class of antibiotics. To address this issue, efforts are being made at multiple levels, including the development of new drugs and practical guidelines to prevent antimicrobial misuse by regulatory agencies. AMR occurs naturally due to spontaneous mutations and the acquisition of resistance genes. As new resistance mechanisms emerge, they quickly spread globally and compromise our ability to treat infectious diseases. A comprehensive understanding of AMR is necessary for developing new tools to measure and predict AMR, rational drug design, and better decision-making by regulatory agencies to combat the spread of antibiotic-resistant bacteria and preserve the effectiveness of antibiotics.

### 3.1. AMR of Pathogens from Farm to Distribution

Monitoring the AMR patterns of clinically important pathogens is also crucial for ensuring the safety of meat products and public health [12]. Previous studies have mainly monitored the emergence of foodborne pathogens from individual farms, slaughterhouses, meat processing plants, or retail stores. In contrast, Bae et al. investigated whether various AMR pathogens in pork meats were transmitted from their production phase (at the farm, slaughterhouse, and meat processing plant levels) to their distribution phase (in retail meat and grocery stores) [8]. Most of the STEC, *L*. *monocytogenes*, and *S. aureus* isolates were resistant to various antibiotics, including ampicillin (AMP), erythromycin, tetracycline (TET), and vancomycin. The most common antimicrobial resistance pattern in the pathogenic STEC isolates was multidrug resistance to AMP, KAN (kanamycin), STR (streptomycin), SXT (trimethoprim-sulfamethoxazole), and TET; however, all of the isolates in the study were susceptible to ciprofloxacin and gentamicin.

### 3.2. AMR of S. aureus Strains Isolated from Human Fecal Samples

*S. aureus* can acquire antimicrobial-resistant genes from other species and transfer them to adjacent bacteria via mobile genetic elements, leading to the emergence and spread of antimicrobial-resistant *S. aureus* strains, such as methicillin-resistant *S. aureus* (MRSA) in healthcare settings [3]. Yoon et al. reported the distribution of antimicrobial resistance and virulence factors in 95 *S. aureus* strains recovered from human stool samples [3]. Only two strains showed no drug resistance, while resistance to three or more antibiotics was observed in 87.4% of strains. AMP resistance was the most common at 90%, and all strains were susceptible to vancomycin.

## 4. Conclusions

In this editorial, we have provided an overview of the articles featured in this Special Issue, which focus on three main topics related to microbial contamination. While we believe that the articles presented in this Special Issue will advance our understanding of microbial contamination, we recognize that there are several other critical topics that have not been covered. These include (1) the quality control of samples, (2) standardization and recommendations for microbial detection and downstream genophenotypic characterization, (3) the use of next-generation sequencing (NGS)-based metagenomics for untargeted detection and genophenotypic characterization of microbial contaminants, (4) artificial intelligence (AI)-based approaches to bridge the gap between genotype and phenotype (such as measuring and predicting AMR using NGS data), and (5) the need for globally integrated system(s) to collect, analyze, and share microbial contamination data across different fields. We hope to gain the opportunity to explore these topics in the near future. Addressing microbial contamination requires a multifaceted approach that involves efforts at various layers interconnected in a pleiotropic and epistatic manner. We express our gratitude to all of the authors who contributed valuable research findings to this Special Issue.
